# Role of Schwann Cells in Preservation of Retinal Tissue Through Reduction of Oxidative Stress

**Published:** 2019-10-01

**Authors:** Alireza Lashay, Raziyeh Mahmoudzadeh, Saeed Heidari Keshel, Asieh Naderi, Roghiyeh Omidi, Fahimeh Asadi Amoli

**Affiliations:** 1 Eye Research Center, Farabi Eye Hospital, Tehran University of Medical Sciences, Tehran, Iran; 2 Department of Tissue Engineering and Applied Cell Sciences, School of Advanced Technologies in Medicine, Shahid Beheshti University of Medical Sciences, Tehran, Iran

**Keywords:** Schwann Cells, Oxidative Stress, Retina, Electroretinogram, ELISA

## Abstract

The aim of this study was to evaluate the effect of subretinal injection of Schwann cells on preservation of retina by decreasing oxidative stress in Dystrophic Royal College of Surgeons (RCS) rats. Schwann cells were harvested from the sciatic nerve of postnatal day 5, RCS rats. Twenty-five RCS rats randomly assigned to cell and sham groups. Schwann cells injected in the sub-retinal space in one eye of the cell group and carrier medium was injected in one eye of the sham group. The proof for the appropriate site of injection of Schwann cells confirmed by the green fluorescent protein (GFP) positive cells. Electroretinogram (ERG) and enucleation for histopathology and enzymatic evaluation were performed 1, 2 and 3 months post-injection. The enzymatic evaluation included catalase, superoxide dismutase (SOD) and glutathione peroxidase 1 (GPx1) by enzyme-linked immunosorbent assay (ELISA) method. Three months after injection, histopathology assessments showed a complete absence of the outer nuclear layer (ONL), photoreceptors and obvious reduction of retinal pigment epithelium (RPE) in the sham group. Cell group showed marked preservation of RPE, choroidal congestion and mild presence of ONL. The green fluorescent protein positive Schwann cells remained in one integrated layer during the study under RPE. The enzymatic evaluation showed that in cell group expression of SOD and GPx1 until month 2 and catalase until month 1 were significantly more than the sham group. At the end of month 3, the amplitude of ERG waves significantly preserved in cell group in comparison to baseline waves and the sham group. We concluded that Schwan cells are able to preserve retinal in RCS rats by reducing oxidative stress.

## INTRODUCTION

A common feature of retinal degenerative disease like retinitis pigmentosa (RP) and age-related macular degeneration (AMD) is early dysfunction of retinal pigment epithelium (RPE) and subsequent loss of rod function which is followed by death of cone photoreceptor cells [1-3]. AMD is the uppermost cause of blindness in elderly and this is gaining more attention because the world is experiencing growth in number and proportion of aged people [4]. It is estimated that 3 million elderly people in the United States will have advanced stages of AMD by 2020 [5]. It is proven that oxidative stress is a major predisposing factor for AMD [6, 7]. Aging and environmental factors like sunlight exposure and smoking, increase oxidative stress [8, 9]. The beneficial outcome of dietary intake of antioxidants supplementation (vitamin C, vitamin E and carotene) and zinc to slow the progression of AMD is shown in several studies [10]. In experimental models, the delivery of growth factors, gene therapy and cell-based therapy can lower the progression rate of AMD and RP [11-14]. A major problem for cell transplantation is the need for immunosuppression because these allogenic cell grafts are prohibited by the host immune system in animal studies [15].

Schwann cells have a critical role in the preservation and renewal of axons of the neurons in the peripheral nervous system (PNS) and secrete different growth factors including glial cell line-derived neurotrophic factor (GDNF) for trophic support of damaged neurons and developing neurons [16]. Schwann cells can support neuronal repair after injury in the central nervous system including spinal cord injury and retinal degenerative disease. Royal College of Surgeon (RCS) rats have an alteration in the receptor tyrosine kinase gene which prevents RPE cells from phagocytosing outer segments of rod cells and results in rod death later [17-20]. RSC rats have normal photoreceptors at birth but changes in photoreceptor nuclei are identified at days 22 and 25 and obvious signs of apoptotic death happen [21]. At day 60 the regular pairing of presynaptic and postsynaptic indicators was completely lost [22].

Syngeneic transplantation is possible for Schwann cells, as they can be harvested and transplanted to genetically identical host and this procedure eliminates the need for immunosuppression [23]. Previous studies have shown that syngeneic subretinal transplantation of Schwann cells can support photoreceptor survival by secreting growth factors such as ciliary neurotrophic factor (CNTF), GDNF and brain-derived neurotrophic factor (BDNF) [24-26]. On the other hand it is shown that Schwann cells can reduce oxidative stress in PNS [27]. So we hypothesized that another mechanism for the supportive role of Schwann cells in the retina can be due to oxidative stress reduction [28].

The aim of this study was to evaluate the role of oxidative stress pathway in retinal degeneration in RCS rats and evaluation of subretinal injection of autologous Schwann cells, using electroretinogram (ERG) and tissue analysis. The Schwann cells were transplanted at an early age before the oxidative stress level was so high to destroy significant numbers of photoreceptors.

## METHODS

**Animals**

Twenty-five pigmented dystrophic RCS rats (Rooyan institute, Tehran, Iran) were used in the study in 2017. Animals were kept in rooms with a 12-hour dark/12-hour light cycle, with ad libitum food and water and constant temperature of 22°C. Animals were kept and handled with the approval of the Institutional Animal Care and an ethical approval was obtained from the Ethics Committee of Tehran University of Medical Sciences. Because the dystrophic changes start from day 22 and complete destroy lasts up to day 120 [21, 22], the enucleation was performed at baseline and at 1, 2 and 3 months after cell transplantation for enzymatic and histopathologic analysis. There were at least 3 rats in each group for different evaluations.

**Preparation of Schwann Cells**

Primary Schwann cells (SCs) harvested from sciatic nerves of 5-day-old RCS rat according to earlier described methods [29, 30]. Sciatic nerves were divided and transferred to Leibowitz L15 medium (Sigma-Aldrich St. Louis, MO, USA)and contaminating tissue removed. The nerves were cut into 100-micrometer (µm) pieces and placed in McIlwain tissue chopper (The Mickle Laboratory Engineering, Brinkmann). The pieces then digested in a mixture of collagenase/trypsin for 90 minutes at the temperature of 37°C. Ten percent fetal calf serum (DMEMF) and Dulbecco modified Eagle medium (Sigma-Aldrich, St. Louis, MO, USA) used for stopping digestion. The digested tissue was centrifuged at 1000 Revolutions Per Minute (RPM) for 5 minutes. After re-suspend and titration the harvested cells were put over poly L-lysine–coated 35-millileter (mm) dishes in DMEMF. Other agents including Amphotericin B, pyruvate, glutamine, penicillin-streptomycin were added and incubated at 37°C 5% CO2. After 24 hours, the medium was changed to eliminate unattached cells and debris. One week later, cells were detached from the dish with trypsin-EDTA and incubated at 37°C in a 100 mm [26] dish coated with rabbit anti-rat IgG beforehand. Cells were ready for transplantation within 24 to 48 hours. All samples were studied using an Invert microscope (CETI fluorescent microscope, UK). Specific markers such as P75 assessed by flow cytometry (Becton-Dickinson, San Jose, CA) and S100 (Sigma-Aldrich,St. Louis, MO, USA), glial fibrillary acidic protein (GFAP) (Sigma-Aldrich,St. Louis, MO, USA) by immunocytochemistry method were used for identification of Schwann cells. The less adherent nature of Schwann cells to the dish and their rapid movement in suspension with distinct morphology made their separation from fibroblasts easy. Finally, syngeneic Schwann cells were collected from dystrophic RCS and marked with a green fluorescent protein (GFP) (Thermo Fisher Scientific) for tracking the cells after injection.

**Transplantation Procedure**

On a postnatal day 21, before the occurrence of any retinal dystrophic changes in RCS rats [21], all animals were anesthetized with the aid of ketamine-xylazine mixture (100/10 mg/kg). One eye of each rat received a subretinal injection of GFP positive Schwann cells (n=10), (3 ×10^4^ cells in 2 microliters [µL] of medium) or it had sham injections (n=10) (2 µL of DMEM + DNase). Grafts were introduced transsclerally into the superior quadrant of the one eye and nonsurgical eyes considered as the control group. Fine glass capillary attached by tubing to a 10-µL Hamilton syringe (Wilmad, Reno, NV) used for subretinal injections. All eyes were examined by indirect ophthalmoscopy (Keeler company, Malvern, PA USA) after injection and any eye showing signs of retinal damage due to injection was eliminated from the study. Anterior chamber (AC) paracentesis was performed before any injection to avoid increase of intraocular pressure.

**Tissue Processing and Histopathologic Study**

Before enucleation at any time (month 1 or 2 or 3 post-injection), the superior edge of the eye was marked. Once animals anesthetized with a maximal dose of ketamine-xylazine mixture (Sigma-Aldrich, St. Louis, MO, USA), enucleation was performed and immediately after that, animals were put in CO box to expire. For assessment of autofluorescence of GFP positive cells, a frozen section (Cryostat, Leica Buffalo Grove, USA) immediately provided from enucleated eye specimens and examined by a fluorescence microscope (Olympus IX81, Tokyo, Japan) armed with a camera. The remained specimens were placed in 10% formalin and incubated overnight at room temperature for fixation. After tissue processing paraffin blocks were prepared and 4-µm tissue sections stained by further Hematoxylin and eosin (H&E) method observed by light microscope (Olympus BX41. Japan). Histopathological study in retina included changes in full thickness of retina including inner and outer nuclear layers (INL and ONL), RPE and photoreceptor layers and ganglion cells layer (GCL).

ERG (Metrovision, France) recordings were performed at 1, 2 and 3-months post injection by the electrophysiological test unit (Metrovision, France). RCS rats were kept in darkness at nights and prepared under faint red light. Before performing the test, animals were anesthetized with the mixture of ketamine and xylazine (100/10 mg/kg). Tropicamide (1%) (Sina-darou, Tehran, Iran) and topical anesthesia (0.5% tetracaine hydrochloride); (Sina-darou, Tehran, Iran) was used to dilate the pupils. To prevent the corneal dehydration, 0.9% saline drops applied regularly on the corneal surface. Goldring recording electrode (4mm, Roland Consult, Brandenburg, Germany), registered the electrical response of the retina. The main recording electrode was set on the corneal surface and the reference electrode was inserted inside the forehead. The ground electrode was positioned on the tail. Eight light stimuli with 125 candela per square metre (cds/m2) were used to record the scotopic ERG. The a-wave and b-wave amplitudes were averaged from three responses with stimulus intervals of 15 millisecond (ms). 

**Enzyme Assessment**

Superoxide dismutase (SOD), catalase and glutathione peroxidase1 (GPX1) are antioxidant enzymes that protect the retina from oxidative damage, and all three enzymes are found in the photoreceptors and in the RPE [31-34]. The enzyme activities were assessed with enzyme-linked immunosorbent assay (ELISA) kits, 1, 2, and 3 months post injection according to the manufacturer’s protocols (Abnova, Taiwan). 

**Statistical Analyses**

Data expressed as mean ± standard error of the mean (SEM). IBM SPSS Statistics (version 24, IBM Corp., Armonk, NY) was used for statistical analyses. A one-way ANOVA, followed by Tukey test was used to identify significant differences between the groups. P value <0.05 was considered statistically significant. All graphs were made by GraphPad Prism software (version 6, GraphPad Software, La Jolla California USA,).

## RESULTS

**Histopathology**

**Figure 1:**
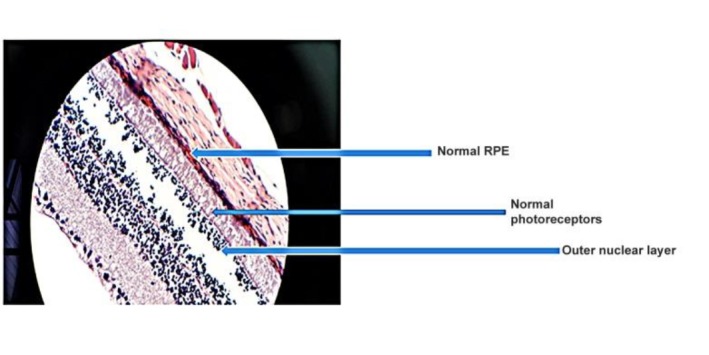
Normal Retina of Royal College of Surgeons (RCS) Rat

**Figure 2:**
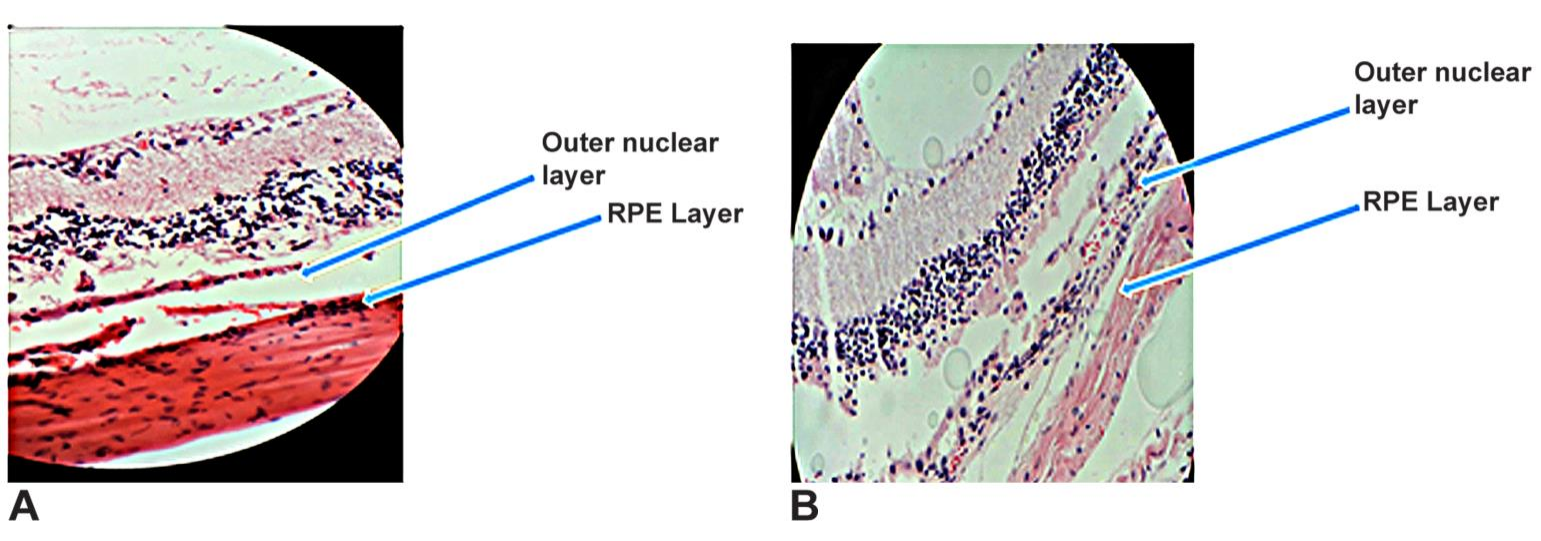
One Month after Injection Retinal Pigment Epithelium (RPE) Reduction is Evident in both Groups but Cell Group has less RPE Reduction. The Left Picture (A) shows the Sham and the Right Picture (B) shows the Cell Groups

The histopathologic features of RCS retina before the start of degenerative process is shown (Figure 1). One month after injection in the cell and sham groups, the RPE layer decreased in both groups but the decrease in cell group was less than the sham group (Figure 2).

**Figure 3:**
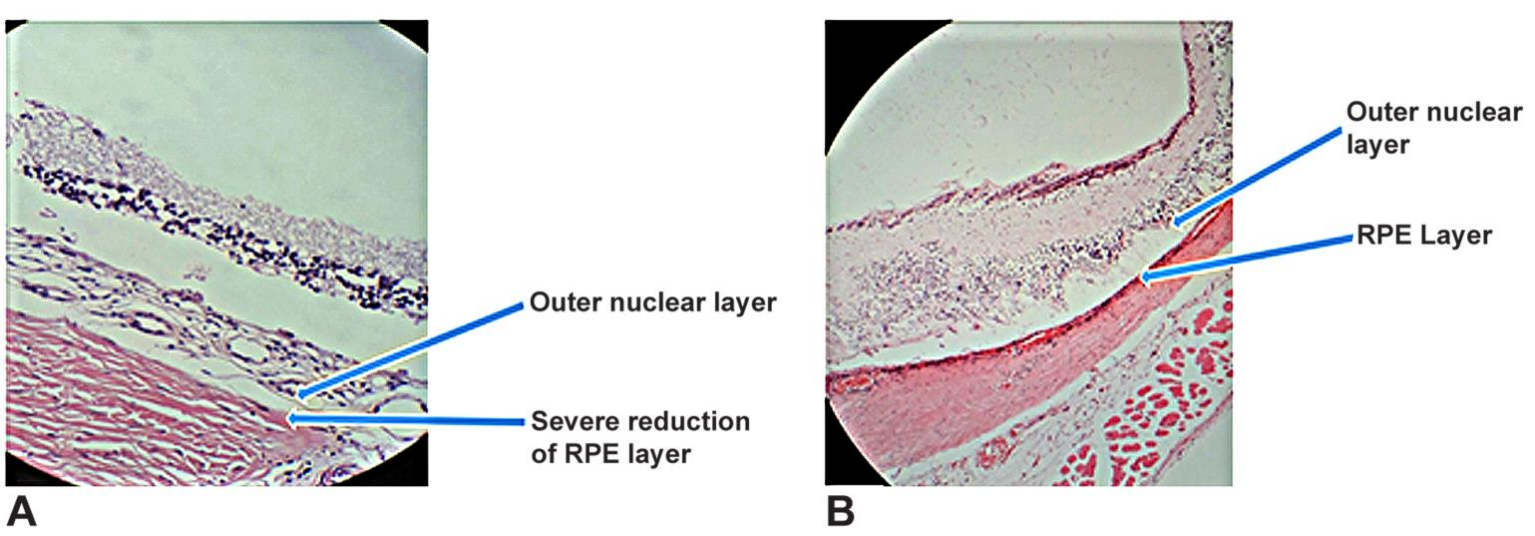
Two Months after Injection. There is outer Nuclear (ONL), Photoreceptors and Retinal Pigment Epithelium (RPE) Layer Reduction in the both Groups but RPE is more preserved in the Cell Group. The Left Picture shows Sham (A) and the Right Picture (B) shows the Cell Groups

**Figure 4:**
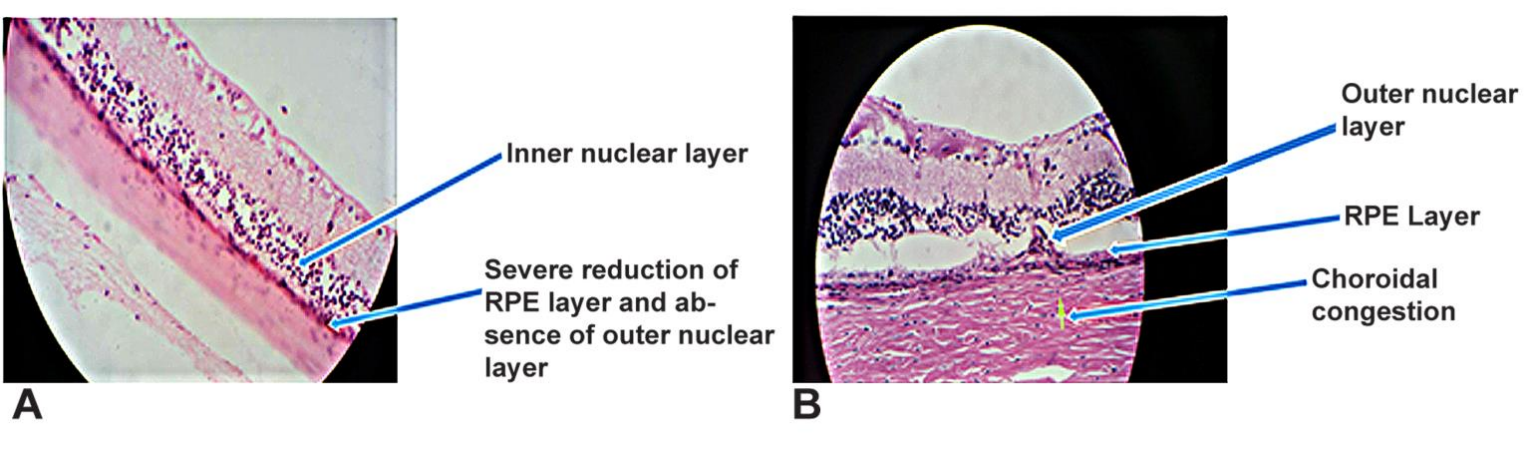
Three Months after Injection. There is a Complete Absence of Outer Nuclear and Photoreceptors Layer along with Obvious Reduction of Retinal Pigment Epithelium (RPE) and Mild Reduction of the Inner Nuclear Layer in the Sham Group (A). Cell Group (B) has a Mild Presence of Outer Nuclear Layer and marked Preservation of RPE Layer associated with Choroidal Congestion

Two months after the injection reduction of ONL, photoreceptors and RPE layer was evident in the both groups but in the cell group, RPE was more preserved (Figure 3). After 3 months, there was a complete absence of ONL and photoreceptors, obvious reduction of RPE and mild reduction of the INL in the sham group, but in the cell group, mild presence of ONL and marked preservation of RPE and choroidal congestion was evident (Figure 4).

**Figure 5:**
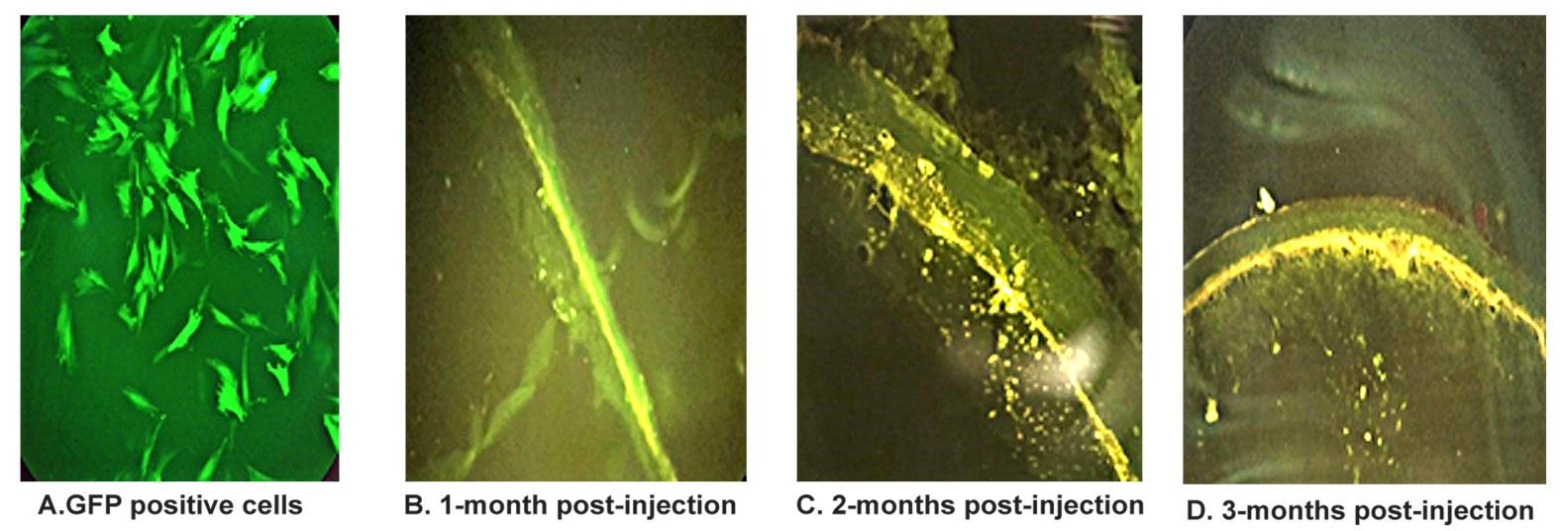
Stability and Integrity of Green Fluorescent Protein (GFP) Positive Schwann Cells after Subretinal Injection in Royal College of Surgeons (RCS) Rats over different Times in Cell Group

The fluorescence microscope showed constant preservation of GFP positive Schwann cells in the retina after 3 months of injection in cell group (Figure 5). GFP positive Schwann cells are shown before injection and after injection as an integrated monolayer of Schwann cells in the retina. These findings indicate that these cells are compatible with retinal tissue.

**Enzyme Assessment**

**Figure 6:**
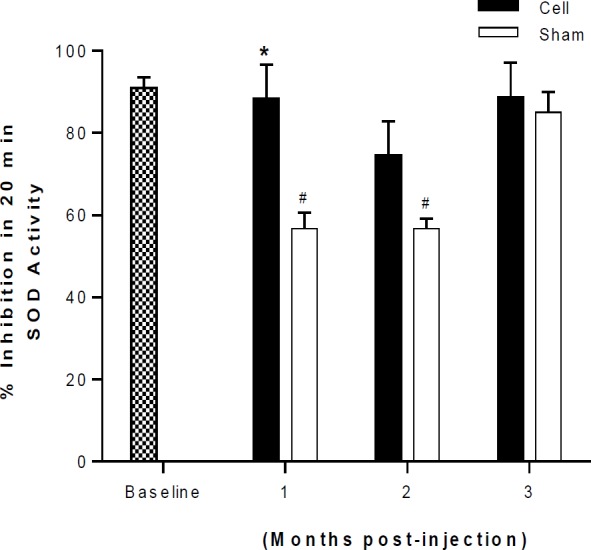
Superoxide Dismutase (SOD) Inhibitional Activity in the Retina of Royal College of Surgeons (RCS) Rats in Cell and Sham Groups following Injection. Data expressed as means ± Standard Deviation (SD). * different from Sham (p-value < 0.05); # different from Baseline, (p-value < 0.05). Note: %: Percentage; min: Minutes; Cell: Cell Group; Sham: Sham Group

SOD activity in the cell and sham groups after injection is shown in Figure 6. One month after injection the sham group (mean ± standard deviation [SD](; 56.66 ± 4.04 %) had significantly lower enzyme activity which is shown based on percentage of inhibition, in comparison to cell group (mean ± SD; 88.33 ± 8.32 %) and baseline group (mean ± SD; 91.00 ± 2.64 %), (P-value =0.00 in both comparisons). In month 2 post-injection both sham and cell groups had lower inhibition activity than the baseline group, but only the sham group (mean ± SD; 56.66 ± 4.04 %) had statistically significant decrease from baseline group (mean ± SD; 91.00 ± 2.64 %), (P-value = 0.01) . In month 3 both cell (mean ± SD; 88.66 ± 8.06 %) and sham groups (mean ± SD; 85.00 ± 5.00 %) showed similar inhibition activities to baseline (91.00 ± 2.64 %) and each other, (P-value = 1.00).

**Figure 7:**
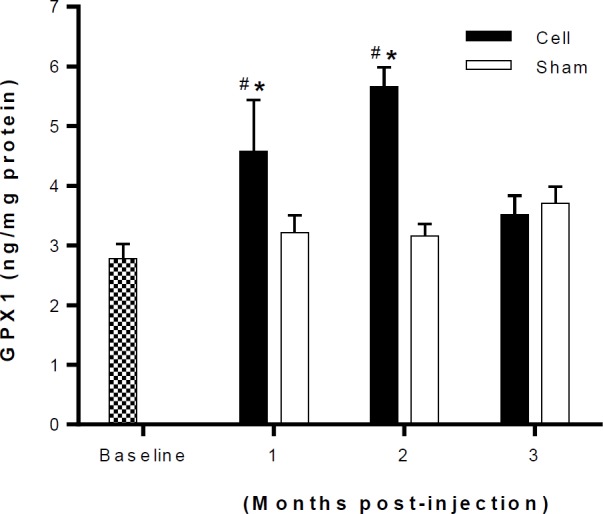
Glutathione Peroxidase1 (GPX1) Activity in the Retina of Royal College of Surgeons (RCS) Rats in Cell and Sham Groups following Injection. Data expressed as Means ± Standard Deviation (SD). * different from Sham (p-value < 0.05); # different from Baseline, (p-value < 0.05). Note: ng/mg: nanogram per milligram; cell: Cell Group; Sham: Sham Group

GPX1 activity in cell and sham groups is shown in Figure 7. One month after injection enzyme level in cell group (mean ± SD; 4.58 ± 0.85 ng/ mg) was higher than sham (mean ± SD; 3.21 ± 0.29 ng/ mg), (P-value = 0.02) and baseline (mean ± SD; 2.77 ± 0.24 ng/ mg) groups, (P-value =0.00). After 2 months the enzyme level increased to (mean ± SD; 5.66 ± 0.33 ng/ mg) in cell group which was significantly higher than baseline (mean ± SD; 2.77 ± 0.24 ng/ mg) and sham group (mean ± SD; 3.16 ± 0.19 ng/ mg), (P-value =.00 in both comparisons). At month 3, cell group enzyme activity (mean ± SD; 3.51 ± 0.32 ng/ mg) decreased to the level of sham (mean ± SD; 3.70 ± 0.27 ng/mg) and baseline groups (mean ± SD; 2.77 ± 0.24 ng/ mg) and there was no significant difference between the groups. 

**Figure 8:**
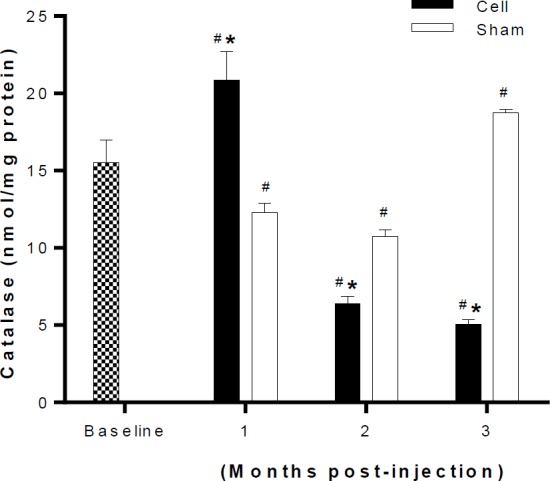
Catalase Activity in the Retina of Royal College of Surgeons (RCS) Rats in Cell and Sham Groups following Injection. Data expressed as Means ± Standard Deviation (SD). * different from Sham (p-value < 0.05); # different from Baseline, (p-value < 0.05). Note: nmol/mg: nanomoles per milligram; cell: Cell Group; Sham: Sham Group

Catalase activity is shown in Figure 8. One month after injection the catalase activity in cell group (mean ± SD; 20.84 ± 1.87 nanomoles per milligram [nmol/ mg]) was significantly higher than sham (mean ± SD; 12.30 ± 0.60 nmol/ mg) and baseline (mean ± SD; 15.53 ± 1.55 nmol/ mg) groups (P-value =0.00 in both comparisons). At month 1 the sham group (mean ± SD; 12.30 ± 0.60 nmol/ mg) also showed significantly decreased activity than the baseline (mean ± SD; 15.53 ± 1.55 nmol/ mg), (P-value = 0.02). Surprisingly in months 2 and 3 the activity level of cell group dropped dramatically and was significantly lower than the sham and baseline groups. In month 2 the cell group activity was (mean ± SD; 6.41 ± .47 nmol/mg) which was lower than sham (mean ± SD; 10.73 ± .45 nmol/mg) and baseline group (mean ± SD; 15.53 ± 1.55 nmol/mg) (P-value=0.002 and 0.00, respectively). At the same month the activity of sham group was also significantly lower than baseline group (P-value=0.001). In month 3, the cell group (mean ± SD; 5.06 ± .29 nmol/ mg) had significantly lower enzyme activity than the sham group (mean ± SD; 18.73 ± .24 nmol/ mg) and baseline group (mean ± SD; 15.53 ± 1.55 nmol/ mg) (P-value=0.00 in both comparisons). The sham group at month 3 (mean ± SD; 18.73 ± .24 nmol/ mg) also had significantly higher enzyme activity than baseline (mean ± SD; 15.53 ± 1.55 nmol/ mg) and cell groups (mean ± SD; 5.06 ± .29 nmol/ mg), (P-value 0.02 and 0.00, respectively).

**Electroretinogram**

**Figure 9:**
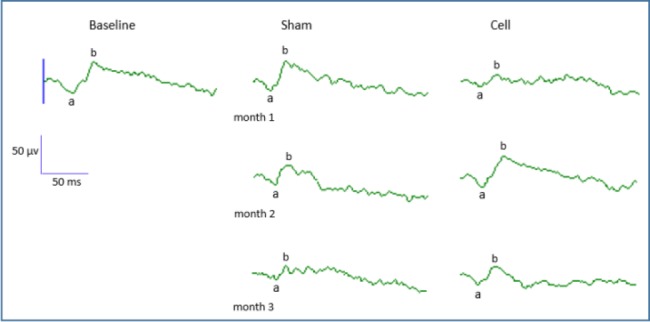
Scotopic Electroretinograms (ERGs) recorded from Royal College of Surgeons (RCS) Rats in Sham and Cell-treated Groups before Transplantation and at Month 1, 2 and 3 after Transplantation. Horizontal Calibration: 50 millisecond (ms). Vertical Calibration: 50 microvolts (μv). Note: a: a-wave; b: b-wave; cell: Cell Group; Sham: Sham Group

The scotopic ERGs were recorded from RCS rats in sham and cell-treated groups before transplantation and at months 1, 2 and 3 after transplantation (Figure 9, Table 1). The changes in amplitude of a wave and b wave in the cell (treatment) and sham group in the injected eye over time is shown in Table 1.

One month after treatment, the a wave was significantly lower in cell group (mean ± SD; -7.06 ± 1.56 microvolts [μv]) in comparison to a wave in baseline group (mean ± SD; -15.95 ± 2.76 μv) (P-value < 0.05). B wave amplitude was also significantly lower (mean ± SD; 10.07 ± 3.63 μv) in comparison to sham (mean ± SD; 28.30 ± 5.46 μv) and baseline groups (mean ± SD; 30.52 ± 4.33 μv) (P-value < 0.05 for both).

In month 2, both a and b waves in cell groups (mean ± SD; -12.73 ± 1.13 μv and 37.43 ± 7.23 μv, respectively) had no statistically significant difference with those of the baseline group (mean ± SD; -15.95 ± 2.76 μv and 30.52 ± 4.33 μv, respectively) and the sham group (mean ± SD; -8.6 ± 2.30 μv and 21.13 ± 7.07 μv respectively) but the cell group had higher amplitude in both a and b waves than sham group and in b wave than baseline. In month 3 the sham group had very significant low amplitude in both a and b waves (mean ± SD; -6.30 ± 1 μv and 8.90 ± 1.20 μv, respectively ) in comparison to baseline (mean ± SD; -15.95 ± 2.76 μv and 30.52 ± 4.33 μv respectively) (P-value < 0.05), but cell group had a higher amplitude in both a and b waves than sham group (mean ± SD; -12.23 ± 3.77 and 18.67 ± 4.01 μv, respectively), which were not statistically different from baseline (mean ± SD; -15.95 ± 2.76 μv and 30.52 ± 4.33 μv, respectively).

**Table 1:** Comparison of a-wave and b-wave Amplitude between Cell and Sham Groups in the Injected Eye

	Baseline	1-month		2-month		3-month	
Groups		**Sham Group**	**Cell Group**	**Sham Group**	**Cell Group**	**Sham Group**	**Cell Group**
a-wave Amplitude (μv)	-15.95 ± 2.76	-9.63 ± 2.91	-7.06 ± 1.56 ^#^	-8.6 ± 2.30	-12.73 ± 1.13	-6.30 ± 1 ^#^	-12.23 ± 3.77
b-wave Amplitude (μv)	30.52 ± 4.33	28.30 ± 5.46	10.07 ± 3.63 ^*#^	21.13 ± 7.07	37.43 ± 7.23	8.90 ± 1.20 ^#^	18.67 ± 4.01

Data are presented as Mean ± SD; n= 3-9 eyes in each group

Abbreviations: n:number; μv: microvolts; SD: standard deviation; * different from sham (p- value < 0.05), # different from baseline (P-value < 0.05).

## DISCUSSION

This study confirmed the previous studies that showed a protective effect of Schwann cells on RCS retina which can be in part due to reduction of oxidative stress level in the retina. The findings were confirmed by enzymatic, electrophysiological and histopathological studies. In 2007 McGill et al. reported that syngeneic subretinal Schwann cell can be a preventive treatment option for retinal degenerative disease. The syngeneic transplantation eliminates the need for immunosuppression and can be harvested easily [30]. The exact mechanism by which Schwann cells can prevent the progression of retinal degenerative disease is unknown but the role of growth factors have been highlighted so far [35]. According to previous studies that showed the ability of Schwann cells to increase the antioxidant defense mechanisms and reduction of oxidative stress in PNS [27], we investigated this pathway in the retina. This study showed that syngeneic subretinal Schwann cell retinal transplantation into the dystrophic RCS rat can preserve the tissue by activating antioxidant enzymes. The ELIZA study showed that enzyme activity is more prominent in the first two months post-injection [postnatal day 81]. Previous studies have shown that degeneration in RCS retina starts early in postnatal day 25 and the most devastating cascades for degeneration are completed at postnatal day 90-120 [21, 22]. We transplanted Schwann cell at postnatal day 21, before the beginning of degeneration process. Enzymatic assessments showed that two months after injection SOD and GPX1 levels were significantly higher than sham and baseline groups. Catalase activity was also significantly higher in the first-month post injection. This finding indicates that up to 90 days postnatal the Schwann cells can still overcome oxidative stress by enhancing enzyme expression but later the cells are unable to stop oxidative stress. 

Pathologic findings showed promising results. The RPE as a critical layer in the retina for the elimination of oxidative stress remained better in cell group. This layer can support the photoreceptors and structure of the retina. The ONL was completely absent 3 months after injection in the sham group but in a cell group, this layer was visible with bridge-like connections with RPE layer. It was shown that Schwann cells can prolong photoreceptor survival time as well [36]. Another important outcome was permanent subretinal presence of Schwann cells which can be a promising finding to show that these cells are likely to be compatible with the retina. Although there are other treatment regimens for retinal degenerative diseases like retinal progenitors derived from embryonic stem cell (ESC) and induced pluripotent stem cell (iPSC), but Schwann cells are immune privilege for retina and secrete different growth factors to rescue the remaining photoreceptors cells [36]. Cell group showed preservation of ONL while the sham group lost ONL at the same time. This feature may help researchers to use Schwann cells as complementary agents for maintenance of retinal structure, secretion of growth factors and reduction of oxidative stress.

ERG results showed a temporary decrease in cell group one month after injection, which might be due to inflammatory response of the retina to newly introduced cells. As time passes, the sham group showed more reduction of ERG waves while the cell group retained better function. This finding helped us to show the better function of retinal layers in the cell group. The ability of Schwann cells to preserve vision was formerly shown by visual behavior tasks, by the aid of optokinetic response (OKR) [30]. 

This study had some limitations including the low number of RCS rats studied and absence of behavioral tests. The strengths of study were understanding of oxidative stress pathways by which Schwann cells can preserve retina and reinforce other pathways to enhance the effect of cell-based therapies. We also suggest further direction by combination of Schwann cells with other cell-based therapies to enhance preventive and therapeutic effects in retinal degenerative disease. 

## CONCLUSIONS

In conclusion our study showed that Schwan cells were able to preserve retina in RCS rats by reducing the oxidative stress pathway. This preservation effect was shown by expression of anti-oxidative enzymes, electroretinogram recordings and histopathological studies.

## DISCLOSURE

Ethical issues have been completely observed by the authors. All named authors meet the International Committee of Medical Journal Editors (ICMJE) criteria for authorship of this manuscript, take responsibility for the integrity of the work as a whole, and have given final approval for the version to be published. No conflict of interest has been presented.

## Funding/Support

None.
